# Mini-transverse incision using a novel bush-hook versus conventional open incision for treatment of carpal tunnel syndrome: a prospective study

**DOI:** 10.1186/s13018-021-02608-x

**Published:** 2021-07-19

**Authors:** Tianxiao Ma, Dongyue Wang, Yuqing Hu, Xiaocui Zhao, Wei Wang, Lihua Song

**Affiliations:** 1 Department of Orthopaedic Surgery, The General Hospital of Jizhong Energy Xingtai Mining Group, NO.202 Bayi Street, Xingtai, Hebei People’s Republic of China; 2 Department of Orthopaedic Surgery, Xiangjiang Area of the Third Hospital of Hebei Medical University, Shijiazhuang, Hebei People’s Republic of China

**Keywords:** Bush-hook, Mini-transverse incision, Carpal tunnel syndrome, Open carpal release, Effectiveness, Safety

## Abstract

**Purpose:**

This study aimed to investigate the outcomes of a mini-transverse incision with a bush-hook versus a conventional open incision for carpal tunnel release (CTR).

**Methods:**

This was a prospective study. The decision to receive either technique (mini-transverse incision with a bush-hook or conventional open incision) was primarily based on patients’ choice. Patients’ symptom severity, functional status, and symptomatic pain were measured at pre-operation, 1 month, and 3 and 6 months postoperatively, and any relevant complications were recorded. Kelly’s scale was used to evaluate the overall clinical efficacy.

**Results:**

Eighty-nine patients were included in the open CTR group and 85 patients in the mini-transverse incision group. The mini-transverse incision group had a significantly smaller incision (4.4±0.6 vs 44.8±3.7 mm), shorter surgical time (7.8±1.9 vs 21.2±3.4 min), and shorter hospital stay (3.7±1.6 vs 5.9±2.0 days) than did the open CTR group. Both groups showed significant improvements from baseline levels (all *P*<0.001). At postoperative 1 month and 3 months, the transverse incision group showed a significantly better VAS, SSS, and FSS (all *P*<0.05), but the difference was non-significant at 6 months except for FSS (*P*=0.022). Also, mini-transverse incision showed a significantly reduced time to return to work and activities, trend to a higher rate of excellence, and good and fewer complications than did the open CTR.

**Conclusions:**

The mini-transverse incision exhibited better performance in surgery-related measures, symptomatic remission, functional recovery, and postoperative morbidity, thus could be considered a promising technique alternative.

**Supplementary Information:**

The online version contains supplementary material available at 10.1186/s13018-021-02608-x.

## Introduction

Carpal tunnel syndrome (CTS) affects 1 to 3.8% of the general population and 5% of the working population who frequently use their hands and wrists in daily activities [1–3]. Surgery is indicated in patients who had no or little response to repeated conservative treatments, e.g., rest, bracing, medications, and local steroid injections. Conventional open carpal tunnel release (CTR) remains the gold standard for surgical treatment of CTS, due to it allowing direct vision of the ligament and surrounding vital anatomic structures. However, the complications directly related to the incision such as cosmetic concern, scar tenderness, pillar pain, and even reflex sympathetic dystrophy might compromise the surgical effectiveness [4, 5]. In contrast, minimally invasive surgery via mini- or limited open incision approach has gained increasingly more popularity due to minimal soft-tissue trauma, less scar or pillar pain, better appearance, and allowing more quickly return to work and daily activities [4, 6–8]. The endoscopic carpal tunnel release (ECTR) technique has been established to reduce incisional discomfort after surgery, but is restricted in a wide use due to the requirement of expensive apparatus, steep learning curve, and the higher risk of neurovascular injury. The mini-incision techniques with variants can offer an easy, quick, and cost-effective alternative to conventional open release or ECTR, despite being a partially “blind” or “semi-blind” procedure [4, 8]. In our practice, we developed a novel mini-profilebush-hook via a 5-mm-length transverse incision at the proximal wrist crease to achieve the purpose of median nerve decompression, with expected theoretic advantages as with other minimally invasive techniques.

By far, the effectiveness and safety of this technique for CTR have not been evaluated. In this study, we aim to introduce this technique and to compare the effectiveness and safety with the conventional open CTR.

## Methods

### Study design and patients

This study was conducted in accordance with Strengthening the Reporting of Observational Studies in Epidemiology (STROBE) guidelines [9]. This was a prospective study, which had been approved by the ethics committee of the General Hospital of Jizhong Energy Xingtai Mining Group, and all the participants had provided written informed consent before it commenced. Between October 2017 and June 2020, consecutive patients who were diagnosed with idiopathic CTS were deemed to be candidates to receive either a mini-transverse incision surgery using a novel bush-hook or a conventional open CTR. Clinical CTS was diagnosed by the presence of at least following 3 findings: clinical symptoms as nocturnal pain or paresthesias, numbness in the median nerve distribution and difficulty with the grasping and use of small objects, physical examinations as Tinel or Phalen signs, and positive electrophysiologic findings via electromyography. The inclusion criteria were definite diagnosis of idiopathic CTS that was limited- or un-responsive to conservative management, e.g., rest, bracing, non-steroidalanti-inflammatory drug (NSAID), injection, physiotherapy for at least 6 months, age at admission < 70 years, and unilateral CTS. The exclusion criteria were history of any surgery at the affected wrist or hand, presence of bilateral CTS, and other causes for CTS such as carpal tunnel tumor, rheumatoid arthritis, cervical spondylosis, or abnormalities in the muscles or tendons of the hand or wrist.

We would explain either procedure including the surgical process, potential benefits, and postoperative complications for each patient, followed by their providing the written content. Patients’ preference was the primary consideration for the decision to perform either procedure. For patients who had no preference for either procedure, the surgical decision was left to the attending surgeon’ discretion.

### Surgical techniques

#### Mini-transverse incision and bush-hook

**Fig. 1:**
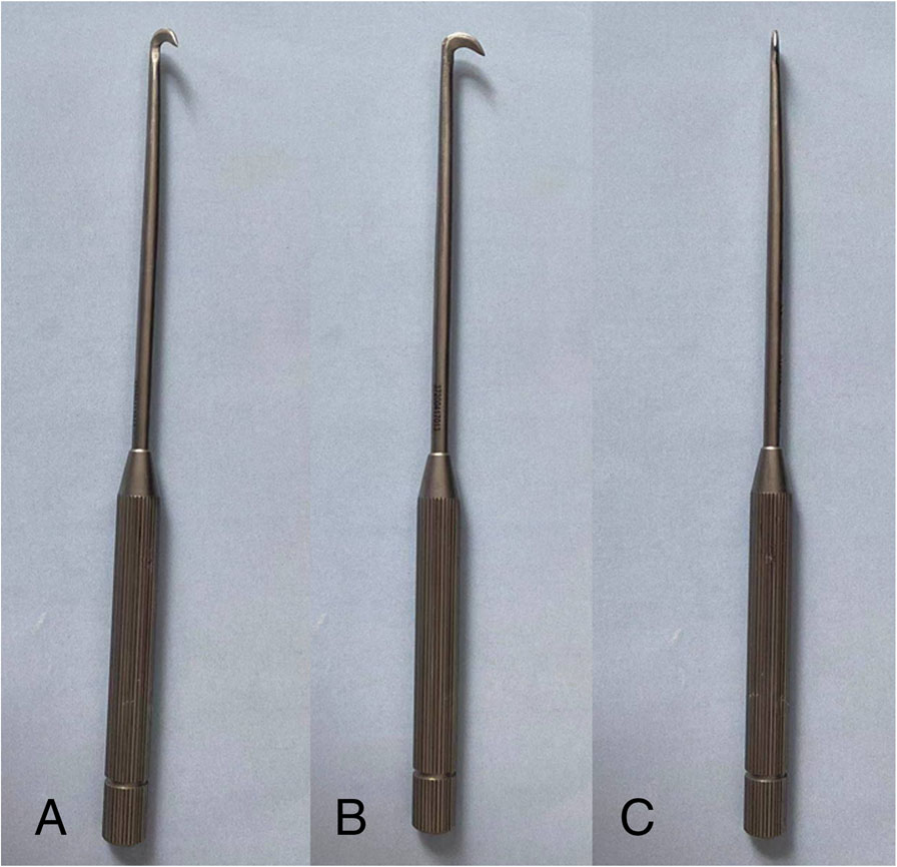
The profile of the mini-bush-hook (**a** oblique view, **b** lateral view, **c** anterior view), 4.7mm in height and 0.8mm in width, featuring the two skids (the tip was blunt) with the sandwiched sickle shaped blade

The mini-profilebush-hook is a knife made of metal entirely, specifically designed for CTR surgery, with measures of 4.7mm in height and 0.8mm in width. It features the two skids (the tip was blunt for purpose of protecting the tissues from cutting trauma) with sandwiched blade between them for cutting the flexor retinaculum for complete release (Fig. 1).

**Fig. 2:**
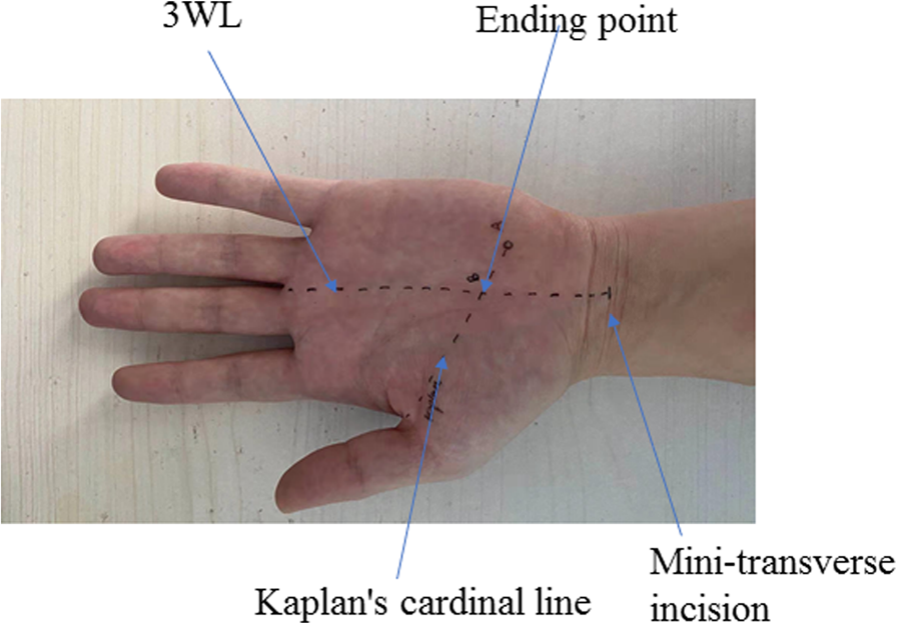
The drawings of the several typical lines or landmarks, mini-transverse at the proximal wrist crease, the 3WL, Kaplan’s cardinal line and the ending point (namely, the intersection point between Kaplan’s cardinal line and extended 3WL)

Under local anesthesia (10–15 ml of 2% lidocaine hydrochloride solution), without tourniquet applied, the patient was in supine position and a padding was placed beneath the affected dorsal wrist to keep it at extension of 25°, with the thumb abducted. A transverse skin incision of about 5mm in length was made along the proximal transverse wrist, with its midpoint located at the intersection point with the 3rd webspace line (3WL), namely the extended line of the web of the ring finger (Fig. 2). A small scalpel or iridectomy scissors was used to dissect the skin and subcutaneous tissues, exposing and opening the aponeurosis of the flexor superficial digitorum tendon. Then, the small-profilebush-hook was introduced into the wrist tunnel and advanced distally along the 3WL, with its tip upwards paralleling to the long axis of the forearm, until to the intersection point of 3WL with Kaplan’s cardinal line (KCL) [10]. At this point, the sudden “give” will be felt, indicating the tip just distal to flexor retinaculum. The bush-hook knife was slowly pulled back proximally to cut the transverse carpal ligament, with the characteristic snap of flexor retinaculum opening being heard; the Freer elevator was then inserted to confirm decompression, and the cut process could be repeated until complete release, if necessary. Careful hemostasis and cleaning of surgical site were done and the skin was closed with 2/0 or 3/0 nylon suture.

#### Open CTR

Under the brachial plexus block (30ml of 0.5% ropivacaine hydrochloride solution) or local anesthesia (10–15 ml of 2% lidocaine hydrochloride solution), the patient was in supine position and a padding was placed beneath the affected dorsal wrist to make it extended at 25°, with the thumb abducted. As described by Taleisnik et al. [11], a palmar longitudinal incision of approximately 3.5 to 4.5 cm in length was made ulnar to the proximal palmar crease, beginning at the axis of the ring finger and passing between the thenar and hypothenar eminences, curved along the axis of the ring finger, and was extended proximally to the proximal flexor crease of the wrist. The subcutaneous tissues were incised and elevated with a retractor to expose the transverse carpal ligament, and median nerve was seen. Then, transverse carpal ligament was incised longitudinally with scissors at the ulnar side of the median nerve. Median nerve entrapment trace could be clearly observed, and if necessary, epineurial release was performed. Careful hemostasis and cleaning of surgical site were done, and the skin was closed with 2/0 or 3/0 nylon suture.

#### Outcome measures

Two investigators (T.M and D.W) evaluated and recorded the outcome measures. Surgical parameters including incision length and surgical duration. Hospital stay was recorded, from the day of admission to discharge. At the postoperative 30 days, namely at the 1st outpatient visit after surgery, the investigators asked the patients whether they returned to the work and activities, and if did, the days since surgery were calculated and recorded; and if no, patients would be asked the such questions at the later visits.

Visual analog scale (VAS) and Boston carpal tunnel questionnaire (BCTQ) were measured at pre-operation, 1 month, 3 months, and 6 months, postoperatively. The BCTQ was a validated self-administered scale and included two subscales: symptom severity subscale (SSS) with 11 items of questions and functional severity subscale (FSS) with 8 items, with a possible score of 1 to 5 for each item; the scoring points for either subscale was determined by the sum of scores divided by the number of items, ranging from 1 to 5 [12]. At the last visit, namely the 6 months after surgery, Kelly’s proposed grading scale was used to determine the overall surgical outcome [13], which was developed based on relief of symptoms for wrist tunnel release. This scale rated the surgical outcome as excellent (complete relief of symptoms), good (persistence of occasional minor symptoms), fair (some constant or annoying symptoms), and poor (symptoms unchanged or worse).

Intraoperative or postoperative complications at each visit were recorded, including injuries to the recurrent motor branch or palmar cutaneous branch of median nerve, superficial palmar arch and adjacent tendons, postoperative surgical site infection, hematoma, scar area pain or pillar pain, or hypertrophic scars.

### Statistical analyses

Shapiro-Wilkes test was used to evaluate the normality of continuous data, which were presented as mean and standard deviation (SD). Paired *t* test was used to evaluate the within-group differences between preoperative and postoperative measurements, namely, the improvements from baselines. Student *t* test or Mann-Whitney*U* test were used to evaluate the between-group differences, as appropriate. Categorical data were presented with number and percentage, and the between-group differences were evaluated using *X*^2^ test. A two-sided alpha level <0.05 was considered significant. All the analyses were performed using SPSS 24.0 (IBM Corporation, Armonk, New York).

## Results

During the study period, 186 patients were enrolled and allocated to the open CTR group (n=98) or the mini-transverse incision group (*n*=88). Nine patients in the open CTR group and three in the mini-transverse incision group were lost to follow-up at different time points. Accordingly, 89 patients in the CTR group and 85 patients in the mini-transverse incision group were included for data analysis. Six surgeons performed all the procedures.

There were no significant differences between the open CTR group and mini-transverse group in demographics (age, 50.4±12.0 vs 48.5±11.8; female predominance, 74.2% vs 72.9%), affected side (right, 51 57.3% vs 54 63.5%), affected dominant wrist (60.6% vs 67.1%), duration of symptoms (20.6±6.5 vs 18.8±7.7 months), or any comorbidities, with all *P* values >0.05 (Table 1). Non-significant difference was also observed for preoperative VAS (3.9±1.6 vs 3.7±1.6, *P*=0.577), SSS score (3.2±0.9 vs 3.4±0.7, *P*=0.673), and FSS score (3.1±0.5 vs 3.2±0.6, *P*=0.704), or the mean follow-up period (6.9±1.1 months in the open CTR group and 6.6±1.2 months in the mini-transverse incision group, *P*=0.618) (Table 2).

**Table 1:** Comparisons of demographics, medical conditions, and comorbidities between mini-transverse

	Mini-transverse incision group (***n***=85)	Open CTR group (***n***=89)	***P***
**Age (year)**	48.5±11.8	50.4±12.0	0.519
**Sex**			0.856
Male	23 (27.1)	23 (25.8)	
Female	62 (72.9)	66 (74.2)	
**Affected side**			0.401
Right	54 (63.5)	51 (57.3)	
Left	31 (36.5)	38 (42.7)	
**Dominance**			0.381
Dominant	57 (67.1)	54 (60.6)	
Non-dominant	28 (32.9)	35 (39.4)	
**Duration of symptoms**	18.8±7.7	20.6±6.5	0.633
**Comorbidities**			
Hypertension	21 (24.7)	25 (28.1)	0.613
Diabetes mellitus	9 (10.6)	11 (12.4)	0.714
Ischemic heart disease	10 (11.8)	10 (11.2)	0.913
Hyperlipidemia	17 (20.0)	21 (23.6)	0.566
Hyperuricemia	11 (12.9)	10 (11.2)	0.730
**Follow-up period (months)**	6.6±1.2	6.9±1.1	0.712

Data presentation: mean ± standard deviation (SD), or number (percentage); *CTR* carpal tunnel release

**Table 2:** Comparisons of surgery-related parameters and clinical results between mini-transverse incision group and open CTR group

	Mini-transverse incision group (***n***=85)	Open CTR group (***n***=89)	***P***
**Incision length (mm)**	4.4±0.6	44.8±3.7	<0.001
**Surgical time (minutes)**	7.8±1.9	21.2±3.4	<0.001
**Hospital stay (days)**	3.7±1.6	5.9±2.0	0.006
**Days to return to work**	8.5±3.9	21.2±5.2	<0.001
**VAS**			
Preoperation	3.7±1.6	3.9±1.6	0.797
Postoperative 1 month	1.2±0.8	1.8±0.8	<0.001
Postoperative 3 months	0.5±0.6	1.0±0.6	0.003
Postoperative 6 months	0.2±0.4	0.5±0.7	0.276
**SSS assessment**			
Preoperation	3.4±0.7	3.2±0.9	0.673
Postoperative 1 month	2.3±0.6	2.6±0.7	0.007
Postoperative 3 months	1.8±0.5	2.1±0.6	0.073
Postoperative 6 months	1.3±04	1.5±0.7	0.394
**FSS assessment**			
Preoperation	3.1±0.5	3.2±0.6	0.904
Postoperative 1 month	2.2±0.8	2.7±0.8	0.003
Postoperative 3 months	1.8±0.6	2.2±0.7	0.022
Postoperative 6 months	1.3±0.5	1.5±0.6	0.466
**Kelly’ grades**			0.304
Excellent	62 (72.9)	56 (62.9)	
Good	17 (20.0)	21 (23.6)	
Fair	6 (7.1)	10 (11.2)	
Poor	0 (1.6)	2 (2.2)	

Data presentation: mean ± standard deviation (SD), or number (percentage); *CTR* carpal tunnel release, *VAS* visual analog scale, *SSS* symptom severity subscale, *FSS* functional severity subscale

With regard to surgical parameters, the mini-transverse incision was associated with better performance, including the smaller incision in length (4.4±0.6 vs 44.8±3.7 mm, *P*<0.001) and shorter surgical time (7.8±1.9 vs 21.2±3.4 minutes, *P*<0.001) than was the open CTR. Hospital stay was significantly shorter in the mini-transverse incision group than in the open CTR group (3.7±1.6 vs 5.9±2.0 days, *P*=0.006) (Table 2).

At postoperative any time points, both groups showed significant improvements from preoperative baseline levels (paired *t* test, all *P*<0.001). At postoperative 1 month and 3 months, the transverse incision group showed a significantly improved VAS, SSS, and FSS (*P* <0.05), except for the SSS (*P*=0.073) that had a better tendency to improvement. At postoperative 6 months, both groups showed the non-significant result (all *P*>0.05). The time to return to the work and activities was 8.5±3.9 days, compared to that of 21.2±5.2 days, being significantly different (*P*<0.001). At the latest visit, the overall rate of excellence and good for mini-transverse incision was 92.9% (62, excellence, 72.9%; 17, good, 20.0%), and for the open CTR was 86.5% (56, excellence, 62.9%; 21, good, 23.6%), with the absolute difference in the percentage of 6.5%, but not being statistically significant (*P*=0.304) (Table 2).

In the open CTR group, scar area pain or pillar pain was found in 3 cases, injury to the motor recurrent branch of the median nerve in 1 case and hypertrophic scars in 1 case. In the mini-transverse incision group, no complications were observed.

## Discussion

Despite that the open CTR remains the gold standard for surgical treatment of CTS, a variety of alternative approaches and novel instruments have been increasingly and widely used in practice and demonstrated to be simple and easy to perform, more importantly allowing a quicker return to the work and activities. In this study, we used the self-developedmini-profilebush-hook via a transverse incision of about 5mm in length for the management of CTS. Compared to conventional open CTR, this minimally invasive technique showed remarkable advantages in operative trauma, early-period symptom relief and functional recovery, and the tendency towards fewer complications. These findings supported this minimally invasive technique as a promising choice in the surgical management of CTS.

There were multiple kinds of mini-incision approaches or novel instruments for CTR surgery [4, 6, 12, 14–19]. The assisted endoscopic technique was one among firstly used techniques, but was limited in wide use for high cost for purchase and maintenance, and the relatively higher rate of iatrogenic injury to vital structures [13]. Without aid of endoscope, Faraj et al. [17] developed two transverse incisions technique and reported the higher satisfaction rate, better cosmetic appearance, and more quick return to the daily activities, compared to the open CTR. In their study, one 1–1.5 cm incision was made at distal wrist crease and second one of 0.5cm at proximal edge of the flexor retinaculum, thus creating a canal through the carpal tunnel for facilitating division of the flexor retinaculum. But the safety and effectiveness of their technique should be validated in large-sample studies, because either group included only 20 subjects. Li et al. [14] used the mini-hook knife combined with dilating metal catheter for wrist tunnel release surgery in their preliminary 12 cases and reported greater theorical safety, which likewise required to be validated in large-sample studies.

KnifeLight via a mini-incision is a most popular technique for CTR and its greatest advantage is the light source emitted in the instrument, making the procedure proceeding under partial vision. This technique combines the advantages of conventional CTR and the endoscopic CTR and its safety and effectiveness have been validated [4, 15, 20]. Nevertheless, the KnifeLight technique did not gain overwhelming popularity, partly due to its relatively large profile with 6.3mm in height and 4.1mm in width for purpose of placing battery and the light source [21]. Therefore, during the process of operation, the tension of tissues around or within the carpal tunnel may be increased especially at the distal part, thereby increasing the risk of morbidity; otherwise, the relatively large incision was required, 12 to 25 mm [6–8]. In this study, the bush-hook was designed with an extremely minimal profile as 4.7mm in height and only 0.8mm in width, thus requiring only a much shorter incision to complete the release. It is of note that, despite being a small procedure, the associated hospital stay was 3.7 and 5.9 days in either group, being significantly different. In fact, this was primarily dependent on the policy of medical insurance reimbursement, because only expenses generated in hospitalization period can be reimbursed.

Compared to the literature studies, our mini-incision technique showed the similar or more favorable results at the postoperative 3 or 6 months. Faraj et al. [17] compared the mini-transverse wrist incision versus traditional longitudinal technique after a mean follow-up period of 3 months and found that the mini-transverse incision group required only 4 days to return to the daily activities and work. Aslani et al. [18] compared regular open incision, mid-palmarmini-incision, and endoscopic technique and found that at postoperative 4 months, the mid-palmarmini-incision and endoscopic technique groups had significantly shorter period to return to work and daily activities (12.1 and 12.7 days) compared to open incision group ( 21.1 days). Wang et al. [16] used three different kinds of small incisions, namely the transverse and longitudinal incision of the wrist, and the longitudinal incisions of the palm to treat CTS, and reported the mean hospital stay was 5.8 days, VAS of 0.6, and 21 days required to return to work. Through the parallel comparisons, our mini-incision technique demonstrated the effectiveness.

There are several important points that should be noted for this mini-incision technique. First of all, we locate the incision at the proximal wrist crease, primarily because this incision is just proximal to distal edge of flexor retinaculum, thus allowing a potential of complete CTR in one-way cut. Furthermore, due to the incision being well hidden within proximal wrist crease, a better cosmesis can be obtained. Secondly, the sudden “give” feeling must be perceived to confirm the distal edge of the flexor retinaculum, and the Freer elevator should be introduced following the first cut to confirm the complete division; and if necessary, second or further cut can be made. Thirdly, operation within the “safe zone” is the key to success of procedure, and the landmarks (3WL and Kaplan’s cardinal line) must be kept firmly in mind to avoid the injury to some vital neurovascular structures (palmar cutaneous branch and motor recurrent branch of median nerve, superficial palmar arch). Additionally, the palmar aponeurosis is preserved so as to reduce the morbidity (tenderness and loss of grip strength), compared to open CTR [22, 23].

The higher risk of scar related complications was always a non-neglectable issue for conventional open CTR procedure [5, 24], and in this study 3, cases of persistent scar tenderness and pillar pain and 1 case of hypertrophic scars were encountered. Also 1 case of injury to the motor recurrent branch of median nerve was encountered, which was presumed to be associated with the variability of the recurrent branch, despite with a low incidence [25]. In contrast, the extremely limited operative canal for the mini-incision technique and being approximately 1 cm ulnar to the median nerve or its branches placed these vital structures at a safer situation.

Several limitations to this study should be noted. Firstly, despite with a prospective design, patients were not randomized to receive either technique, and this non-randomization would have biased the results. Secondly, we selected 6 months as the follow-up endpoints, for it is a reasonable period for symptom relief and functional recovery. Our results that were similar in SSS and FSS between two groups also demonstrated the reasonability. Thirdly, a total of 6 surgeons with differentiated surgical skill level performed the procedures, but due to the limited subject sample for each surgeon, the role of surgeon experience cannot be defined. Fourth, we did not capture the data on the types of work for the included patients, and it is possible that the type of work would affect the time to return to work. But considering the very large gap (8.5±3.9 vs 21.2±5.2 days) on this outcome measure between two groups, we thought it was unlikely to use the type of work to explain. Fifth, this is a single-center study which might limit the generalizability of the results, and our findings require to be validated in multi-center studies.

In summary, we introduced a novel mini-profilebush-hook via a mini-transverse incision at the proximal wrist crease for CTR. This mini-incision technique exhibited better performance compared to the conventional open CTR, with regard to remarkably shorter incision, shorter surgical duration, more improved early-period symptom relief and functional recovery, more quickly return to work and activities, and fewer complications. This mini-incision technique via mini-profilebush-hook could be considered in practice as a promising alternative to conventional open CTR technique.

## Data Availability

All the data will be available upon motivated request to the corresponding author of the present paper.

## Addiitonal files

**Additional file 1 **: **Video 1**. **Additional file 2 **: **Video 2**.

## Abbreviations

CTR: Carpal tunnel release
CTS: Carpal tunnel syndrome
ECTR: Endoscopic carpal tunnel release
NSAID: Non-steroidal anti-inflammatory drug
3WL: 3rd webspace line
KCL: Kaplan’s cardinal line
VAS: Visual analog scale
BCTQ: Boston carpal tunnel questionnaire
SSS: Symptom severity subscale
FSS: Functional severity subscale
SD: Standard deviation
