# Correction to: Mini-transverse incision using a novel bush-hook versus conventional open incision for treatment of carpal tunnel syndrome: a prospective study

**DOI:** 10.1186/s13018-021-02712-y

**Published:** 2021-09-14

**Authors:** Tianxiao Ma, Dongyue Wang, Yuqing Hu, Xiaocui Zhao, Wei Wang, Lihua Song

**Affiliations:** 1 Department of Orthopaedic Surgery, The General Hospital of Jizhong Energy Xingtai Mining Group, No.202 Bayi Street, Xingtai, Hebei People’s Republic of China; 2 Department of Orthopaedic Surgery, Xiangjiang Area of the Third Hospital of Hebei Medical University, Shijiazhuang, Hebei People’s Republic of China

**Correction to: J Ortho Surg Res. 16, 462 (2021)**

**https://doi.org/10.1186/s13018-021-02608-x**

Following the publication of the original paper [1], the authors found out that the affiliations have been incorrectly captured. Updated information is provided in the affiliation section above.

The original article has been corrected.

## References

[1] Ma T, Wang D, Hu Y, Zhao X, Wang W, Song L (2021). Mini-transverse incision using a novel bush-hook versus conventional open incision for treatment of carpal tunnel syndrome: a prospective study. J Orthop Surg Res.

